# Process Evaluation of a Randomized Controlled Trial of Group Support Psychotherapy for Depression Treatment Among People with HIV/AIDS in Northern Uganda

**DOI:** 10.1007/s10597-017-0129-4

**Published:** 2017-03-19

**Authors:** Etheldreda Nakimuli-Mpungu, Kizito Wamala, James Okello, Sheila Ndyanabangi, Steve Kanters, Ramin Mojtabai, Jean B. Nachega, Edward J. Mills, Seggane Musisi

**Affiliations:** 1 College of Health Sciences, Makerere University, Kampala, Uganda; 2 Center for Victims of Torture, Gulu, Uganda; 3 Department of Psychiatry, Gulu University, Gulu, Uganda; 4 Ministry of Health of Uganda, Kampala, Uganda; 5 Precision Health Economics, Vancouver, Canada; 6 Department of Mental Health, Johns Hopkins Bloomberg School of Public Health, Baltimore, USA; 7 Faculty of Medicine and Health Sciences, Stellenbosch University, Cape Town, South Africa; 8 Department of Clinical Epidemiology & Biostatistics, McMaster University, Hamilton, Canada; 9 Department of Psychiatry, School of Medicine, College of Health Sciences, Makerere University, P.O. Box 7072, Kampala, Uganda

**Keywords:** Randomized trial, Group support psychotherapy, Process evaluation, Depression, Persons living with HIV/AIDS, Uganda

## Abstract

We describe the process evaluation for a randomized controlled trial that compared group support psychotherapy (GSP) with group HIV education for treatment of depression among people with HIV. Process data were obtained using mixed methods. Variables evaluated were indicators of feasibility and acceptability; causal mediating processes and contextual influences. GSP was feasible and acceptable. Potential mediating variables between GSP and reduction of depression were improved emotional and social support, better coping strategies, and pursuit of livelihoods. Culturally sensitive intervention content facilitated intervention delivery. These data complement the trial outcomes, and may provide a contextualized description of how GSP treats depression.

## Introduction

Depression in people living with HIV (PLWH) was recognized early on in the AIDS epidemic as a key factor affecting HIV treatment outcomes in developed countries (Brown et al. 1992; Lyketsos et al. 1996). Yet, it is only in recent years that attention has focused on this issue in developing countries (Marwick and Kaaya 2010; Chibanda et al. 2011; Nakimuli-Mpungu et al. 2013a). A recent systematic review of studies documenting the prevalence of depression in HIV treatment programs across sub-Saharan Africa indicated that one in three PLWH had significant depression symptoms (Nakimuli Mpungu et al. 2012).

The concern regarding co-morbid depression is not only regarding its effect on motivation to adhere to antiretroviral therapy (ART) (Uthman et al. 2014; Nakimuli-Mpungu et al. 2012) but also, its association with behaviors that may facilitate HIV transmission (Musisi et al. 2014). Further, depression has been associated with failure to access HIV care and treatment (Pence et al. 2012), increased morbidity (Ironson et al. 2005; Hartzell et al. 2008) and mortality (Leserman et al. 2007). The growing recognition that HIV associated depression needs to be tackled if HIV prevention, treatment and care programs are to be successful has led to an expanding body of knowledge in recent years based on randomized trials of psychological interventions for depression among PLWH in developing countries (Chibanda et al. 2015).

In Uganda, to address the problem of depression in PLWH, we developed a culturally sensitive group support psychotherapeutic (GSP) intervention to be used as first line treatment for depression in rural primary care settings. We used a randomized controlled trial to assess the specific effects of GSP by comparing it with an active comparison, group HIV education (GHE), directly after treatment and after 6 months. GHE was modelled after existing programmes that credibly aim to generate therapeutic benefits in common with GSP without the specific elements of GSP (Nakimuli Mpungu et al. 2015).

We hypothesized that GSP would lead to acquisition of knowledge and skills that enhance their social connections and support and help them cope better with adverse situations and stigma. These changes would lead to a reduction in depression symptoms. The absence of depression would improve ability to work and obtain savings and other livelihood assets. The pursuit of livelihoods would help restore the dignity and independence of PLWH, thereby leading to a further reduction in stigma, which, in turn, would sustain reduction in depression symptoms and improvement in functioning. In keeping with our hypothesis, GSP was more effective than GHE in reducing depression symptoms and increasing functioning levels in patients with HIV in the long-term (Nakimuli Mpungu et al. 2015).

In this paper, we provide data from the process evaluation of this trialwhich compared group support psychotherapy (GSP) with group HIV education (GHE) for treatment of depression in people with HIV in northern Uganda. The collection of process data was informed by Linnan and Steckler’s process evaluation frameworks (Linnan and Steckler 2002). Specifically, we aimed to evaluate indicators of feasibility, acceptability, attrition, and fidelity. We also aimed to clarify our hypothesized causal mechanisms and explore the contextual influences on intervention delivery and outcomes.

## Methods

### Study Setting

Kitgum district is part of the post-conflict northern region of Uganda. Over 90% of the population is engaged in small-scale agriculture and animal husbandry as their major income generating activity. The civil war that the people of Kitgum endured for two decades (1987–2007) led to a breakdown of health care delivery systems, and loss of property and infrastructure (Atekyereza 2015).

## Overview of the Trial

This was an open-label randomized controlled trial that included men and women with HIV, aged 19 years or older from an urban HIV care centre in Kitgum district, northern Uganda, who met the Mini International Neuropsychiatric Interview criteria for major depression. Participants were randomly assigned to receive 8 weekly sessions of either GSP (n = 57) or GHE (n = 52). Randomization was achieved by allowing men and women separately to pick pieces of paper containing the intervention allocation code from a basket (1:1 allocation ratio). The intervention sessions were provided in gender-specific groups. Participants were followed up immediately after the intervention and again 6 months after the end of treatment. The primary outcomes were change in depressive symptom scores (measured with the Self-Reporting Questionnaire) and in functioning scores (measured with a locally developed method). The data were analyzed following an intention to treat approach using cluster-adjusted *t* tests and permutation tests.

 This trial was registered with The Pan African Clinical Trials Registry, number PACTR201402000742370. The study was submitted to and approved by both the Makerere University College of Health Sciences Research Ethics Committee and the Uganda National Council of Science and Technology. All study participants provided written informed consent. Light refreshments were served during all group sessions in both treatment groups and every participant received an equivalent of US$2–5 to defray transport costs. Full details of the trial are described elsewhere (Nakimuli-Mpungu et al. 2015).

## The Interventions

### Group Support Psychotherapy (GSP)

The development of GSP involved the review of the pre-existing group counseling in psycho-trauma centers within regional referral hospitals in northern Uganda (Nakimuli-Mpungu et al. 2013b) and focus group discussions with community members to identify community perceptions of depression, local strategies used to deal with depression, community experiences with group interventions and opinions on what would be the most culturally acceptable components of a group support psychotherapy intervention to alleviate depression symptoms in HIV-affected adults. On the basis of the findings from these two activities, a manual for implementation of the 8-week group support psychotherapy intervention was developed by the investigating team. Details of the development process and the structure of the intervention have been described elsewhere (Nakimuli-Mpungu et al. 2014a).

GSP was delivered in eight sessions held weekly, lasting 2–3 h each. Participants were divided into gender specific groups of 10–12 participants. Intervention facilitators were mental health workers with a mental health diploma or degree, of the same gender as the participants, and they delivered the intervention material following a scripted intervention manual (MHIN 2014).

### Group HIV Education (GHE)

The GHE intervention was designed and delivered in a similar format as the GSP intervention. We ensured structural equivalence by including the same number of sessions as those of GSP (eight 2-h sessions run on a weekly basis by one facilitator and similar group sizes, a mental health worker with a diploma or degree, of the same gender as the group members, and who delivered the intervention materials following a scripted intervention manual). The GHE manual has been described elsewhere (Nakimuli-Mpungu et al. 2015). Briefly, it was developed with the goal in mind to create a comparison group intervention whose rationale and procedures would be credible enough to generate therapeutic factors in common with GSP such as a supportive environment, therapeutic alliance, and therapeutic optimism while not including the active elements of GSP such as opportunity to express emotions, and acquisition and practice of positive coping skills and livelihood skills. Table 1 summarizes the content of GSP and GHE group sessions.

**Table 1:** The content of group support psychotherapy and Group HIV education group sessions

	Group support psychotherapy	Group HIV education
Session 1	Group facilitators (GFs) assist in building group cohesion, explain the therapeutic process of GSP, guide the group members (GMs) as they lay the ground rules and elect a group leader among them GFs explained how GSP will heal depression and reassure GMs that the GSP intervention will provide a safe and supportive environment in which confidentiality is maintained GMs are asked to make a commitment to attend all group sessions	The first session of GHE focuses on introductory issues, building group cohesion, explaining the rationale of HIV education. GFs guide GMs as they lay the ground rules and elect a group leader among them
Session 2	The GFs educate GMs about depression and its relationship with HIV/AIDS, the presentation, triggers, complications and treatment options for depression and help GMs understand the link between HIV and depression The impact of depression on adherence to antiretroviral medications is emphasized in this session	In the second session, GFs educate about the progression of HIV in the body. Emphasis is placed on explaining the four stages of HIV infection that most people are asymptomatic and the virus can be transmitted to others at any stage of HIV infection. Also, the difference between HIV and AIDS is explained
Sessions 3 and 4	GFs encourage GMs to share their personal problems with others, to seek support and to receive and provide feedback The GF engages the group in a discussion on the pros and cons of currently used coping strategies to deal with their personal problems	The third and fourth sessions the GFs educates about the various ways in which HIV may be transmitted from one person to another transmission and prevention of HIV infection
Session 5	GFs highlight the depressive thinking and excessive worries that GMs demonstrated as they shared their personal problems GFs teach GMs how to identify positive and negative ways of thinking and how to replace negative thoughts with positive thoughts GFs also demonstrate how to positively cope with excessive worries usually experienced when one is going through tough situations	The GFs educate about mother-to-child transmission of HIV and explain specific ways in which the HIV virus may be transmitted from a mother to her child
Session 6	GFs teach GMs problem solving skills and how to cope with stigma at personal, family, and community level	The GFs continue to educate about mother-to-child transmission of HIV and explain specific ways in which transmission of the HIV virus from a mother to her child may be prevented
Sessions 7 and 8	In session 7, GFs illustrate basic livelihood skills that will enable GMs to identify income generating activities that will improve their livelihoods thus enabling them to take control of their lives GFs guide discussions on what small viable enterprises GMs may engage in together or in smaller groups using their own resources In session 8, GMs are asked to demonstrate basic livelihood skills learned by allowing them to present their business ideas to the group. Discussions are held about various business ideas presented by the GMs	The last two sessions focused on basic facts about antiretroviral therapy (ART) with emphasis on side effects, drug interactions and the importance of ART adherence to prevent emergence of drug resistance

### Training of the Mental Health Workers

Prior to the delivery of GSP, mental health workers working in the Kitgum mental health clinic were trained to facilitate GSP group sessions in a 5-day competency based training workshop led by psychiatrists and clinical psychologists from Makerere University. The goals of the workshop were to enhance knowledge and skills in four areas of competence including initial contact and rapport building, screening for depression and triage, delivery of GSP using the GSP manual, and referral of non-responsive cases. To achieve these goals, the workshop facilitators used active learning techniques, involving role playing, brainstorming sessions, problem-solving games, and small group discussions. Key role-plays and sessions in which skills were demonstrated were videotaped and recordings were provided to all trainees to assist them with continuing review of skills demonstrated. All trainees participated in pilot GSP sessions (Nakimuli-Mpungu et al. 2014b) before delivering group sessions in the trial.

### Study Measures

#### Assessment of Feasibility

Feasibility was assessed by recording the proportion of PLWH randomized to receive either GSP or GHE who received either intervention (*Reach*), and the proportion of PLWH who attended six or more group sessions of either intervention (*dose delivered*). The proportion that was lost to follow-up (*attrition*) was determined from the attendance registers provided by GFs following assessments at the end of treatment and at 6 months follow-up.

#### Assessment of Acceptability

Acceptability was assessed using a mix of qualitative and quantitative measures. A self-administered semi-structured questionnaire completed by GFs after the end of each group session included questions regarding acceptability of content to GMs. Research assistants administered an eight-item questionnaire adapted from measures previously used to evaluate interventions targeting mental health issues of PLWH (Purcell et al. 2007). The items assessed satisfaction with intervention content, GFs’ knowledge and attitudes. GMs were asked to indicate the extent to which they agreed with the items. Responses were based on a four-point Likert scale with higher scores indicating greater satisfaction.

#### Assessment of Fidelity

We designed a self-administered semi-structured questionnaire to be completed by GFs after each group session. They were required to describe content delivered in each group session and send their responses to mental health specialists who had trained them via e-mail. For any problematic issue raised, advice was given over the phone on how to address the issue. The GFs responses gave an indication as to whether the intervention delivery had progressed as planned or not.

#### Assessment of Causal Mechanisms and Contextual Influences

**Fig. 1:**
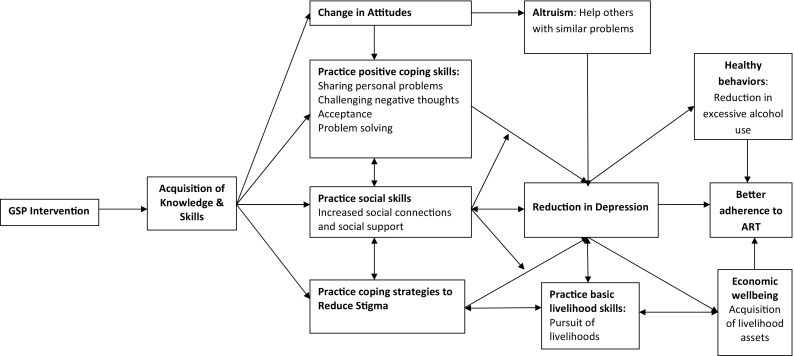
Qualitative model depicting the causal pathways between the GSP intervention, reduction in depression and outcomes of depression treatment

To explore our hypothesized causal mechanisms illustrated in Fig. 1, GFs used the same self-administered semi-structured questionnaire, to report on perceived GMs changes in knowledge, skills, attitudes and behaviors as well as contextual factors that may have facilitated or hindered the intervention delivery process.

### Stigma

To measure enacted stigma, we used a total of 13 items adapted from an HIV/AIDS indicator survey previously used by Nyablade and Mac Quarrie (2006) which described various forms of discriminatory events experienced by PLWH as a result of their HIV status.

### Adherence to Anti-retroviral Therapy

The questionnaire also included one item to assess adherence to anti-retroviral therapy (ART) to assess any changes in adherence associated with the reduction in depression. GMs were asked to report the number of days on which they had missed any dose of ART in the week prior to the 6 month follow up.

### Statistical Analysis

For the qualitative data, interview transcripts from GMs and the GFs were reviewed for accuracy and were then transcribed verbatim before translation into English. To control for errors in translation, two research assistants fluent in English and the local language (Luo), worked together with the third author (JO) to translate and electronically transcribe the data (Drennan et al. 1991). QRS Nvivo 10 qualitative data analysis software was used for coding and thematic analysis (Braun and Clarke 2006).

The interview data were initially coded according to a number of themes that corresponded to the focus questions. The codes were used to construct matrix displays based on the co-occurrence of codes and the two treatment groups. The resulting matrix display provided both the frequency of responses and the detailed content of responses, allowing us to assess how often responses varied between GFs and GMs as well as between the two treatment groups. Inter-coder reliability was not determined.

For the quantitative data, we conducted bivariate analyses with χ^2^ tests and independent *t* tests to compare baseline demographic variables between the GSP and GHE participants who participated in the process evaluations 6 months after the end of treatment. Although randomization to GSP and GHE was done at the individual level, study participants received their respective interventions in groups (clusters). Thus, to account for the clustered nature of the data, we applied the STATA *clttest* command to obtain cluster-level summaries and also used permutation tests which is a robust method of analysis for clustered data (Hayes and Moulton 2009). To evaluate stigma, factor analyses of the stigma sale were conducted.

## Results

### Characteristics of Study Participants

A total of 109 PLWH participated in the trial. Their baseline socio-demographic characteristics are described in detail elsewhere (Nakimuli-Mpungu et al. 2015). Six months after the end of treatment, 85 of these participants returned for follow-up assessments with interviewer-administered semi-structured questionnaires. Their socio-demographic characteristics are summarized in Table 2. Ten group facilitators completed self-administered semi-structured questionnaires at the end of each group session and four of these individuals were interviewed for 15 min 6 months after the end of treatment. Their average age was 32 years (range 28–41), and 40% were males.

**Table 2:** Baseline socio-demographic characteristics of trial participants 6 months after treatment

	Group support psychotherapy (n = 45) N (%)	Group HIV education (n = 41) N (%)
Age (years) Mean (SD)	40.53 (1.23)	41.04 (1.40)
Gender		
Female	30 (67)	23 (56)
Male	15 (33)	18 (44)
Educational background		
Primary education or lower	38 (84)	32 (78)
Secondary education or higher	7 (16)	9 (22)
Occupational status		
Not employed	11 (24)	11 (27)
Employed	9 (20)	13 (71)
Peasant farmer	25 (56)	17 (42)
Relationship status		
Single	4 (8)	3 (7)
Married	16 (36)	21 (51)
Separated	4 (9)	4 (10)
Widowed	21 (47)	13 (32)
Number of children (Mean (SD))	4.5 (2.1)	4.8 (3.1)

### Indicators of Feasibility

Of 57 participants assigned to GSP and 49 assigned to GHE, 50 (88%) and 49 (94%) participated in group sessions respectively, while 46 (81%) and 49 (94%) attended 6 or more sessions respectively, suggesting that the reach of the intervention, dose delivered and received were favorable. Although the reach was comparable between the two groups (χ^2^ = 1.38, p value = 0.24), attendance was significantly more frequent in the GHE than GSP group (χ^2^ = 4.5, p value = 0.03). Attendance was significantly associated with gender and marital status, with male participants (OR = 0.24, p value = 0.03) and unmarried individuals (OR = 0.13, p value = 0.05) less likely to attend six or more sessions. Reasons for missing sessions included poor physical health, conflicting work schedules, and burial attendances.

Attrition was similar in the two groups, with 13 (23%) GSP and 11 (21%) GHE participants unavailable for 6-month follow-up assessments. Except for hazardous alcohol consumption (higher in non-completers than in completers), participants lost-to-follow-up did not differ significantly on other baseline and psychosocial variables from those who completed study assessments (Nakimuli-Mpungu et al. 2015). Reasons for attrition were unknown for seven participants; for others, they included death of the group participant or death of a household member necessitating a burial attendance, migration to South Sudan or neighboring districts, hospitalization, and imprisonment.

### Indicators of Acceptability

Although GFs reported poor time management especially in the initial sessions, the GMs continued to attend group sessions with keen interest and active participation. During the GSP sessions 3 and 4 where GMs shared their painful personal experiences, GFs reported that they were surprised by the passion and enthusiasm with which these sessions were embraced by the participants. A male GF commented:

“The members who shared their personal problems were thorough and explicit; while the other members were cooperative as they came in to support them.” (Male GF for GSP sessions).

Although both GSP and GHE participants expressed a keen interest in the intervention content, their evaluation of the interventions indicated that GSP participants were more satisfied with their intervention than GHE participants: Table 3.

**Table 3:** Participant evaluation of group support psychotherapy and group HIV education interventions

Cluster level summaries	Group support psychotherapy N = 5	Group HIV education N = 5	Permutation test	
			Estimated effect*	P value (95% CI)
Immediately after treatment				
I have learned a lot from this intervention	2.82 (0.04)	2.65 (0.04)	0.17	0.09 (0.08–0.09)^†^
I would recommend this intervention to other people	2.78 (0.04)	2.66 (0.04)	0.13	0.24 (0.23–0.24)
Group leaders were knowledgeable	2.69 (0.11)	2.48 (0.11)	0.21	0.22 (0.21–0.23)
Group leaders were supportive	2.62 (0.07)	2.57 (0.07)	0.06	0.65 (0.64–0.66)
Group leaders were respectful	2.70 (0.08)	2.61 (0.08)	0.09	0.41 (0.40–0.42)
This intervention reduced my depression	2.72 (0.05)	2.49 (0.05)	0.23	0.05 (0.05–0.06)
Helped me to talk to other people about what we learned	2.60 (0.07)	2.41 (0.07)	0.18	0.14 (0.14–0.15)
Intervention motivated me to make positive changes in my life	2.74 (0.08)	2.52 (0.08)	0.22	0.08 (0.07–0.08)^†^
6 months after the end of treatment				
I have learned a lot from this intervention	2.80 (0.08)	2.63 (0.08)	0.16	0.17 (0.16–0.19)
I would recommend this intervention to other people	2.83 (0.10)	2.57 (0.10)	0.26	0.08 (0.07–0.09) ^†^
Group leaders were knowledgeable	2.80 (0.04)	2.74 (0.04)	0.06	0.39 (0.37–0.41)
Group leaders were supportive	2.76 (0.06)	2.81 (0.04)	0.05	0.61 (0.59–0.61)
Group leaders were respectful	2.87 (0.05)	2.86 (0.05)	0.01	0.95 (0.94–0.96)
This intervention reduced my depression	2.85 (0.11)	2.41 (0.11)	0.42	0.01 (0.01–0.02)
Helped me to talk to other people about what we learned	2.84 (0.14)	2.49 (0.14)	0.34	0.14 (0.12–0.15)
Intervention motivated me to make positive changes in my life	2.84 (0.14)	2.49 (0.14)	0.34	0.14 (0.12–0.15)

Data are Mean (SE)

*The mean difference between group support psychotherapy and group HIV education

^†^Marginal significance

### Indicators of Fidelity

All GFs in both groups provided feedback on what transpired during each group session indicating that all planned sessions were delivered. However, in some instances, the content delivered deviated from what was stipulated in the manuals. In the GSP groups, GFs responses indicated that the intervention content was delivered more or less as planned except for the session on positive coping skills. Introduction of this session was challenging across all GSP groups because GMs wanted to continue with sharing their painful personal problems, an activity which should have been completed in sessions 3 and 4. Consequently, session 5 started with sharing of personal problems in the first hour after which the GFs introduced the session on positive coping skills.

In the GHE sessions, the mode of delivery of education sessions deviated from what was stipulated in the manual. The education sessions were to be delivered using a lecture format with a question and answer session at the end of the lecture with minimal interaction and discussion. However, GFs responses indicate that the education sessions were interactive with lots of discussions and with some GMs insisting on sharing their experiences. One GF of GHE sessions commented:

“Some members want to share more of their personal stories and problems and yet this group is only to increase their knowledge of HIV/AIDS. However, they were guided and asked to seek individual counseling to help them overcome their problems.” (Female GF for GHE sessions).

### Hypothesized Causal Mechanisms

On the whole, PLWH began the interventions with very little knowledge, poor time management and skepticism about the intervention’s ability to reduce their depression. However, after the GFs explained how the group process would help them, and what role was expected of them in order to recover from depression, the GFs observed better time management, keen interest and active participation in all group activities by the GMs. During the GSP sessions in which GMs shared painful personal experiences, the GFs reported that they were surprised by the passion and enthusiasm with which these sessions were embraced by the GMs. The positive feedback from GMs and the observed positive changes in their emotions and behavior after these sessions removed all skepticism, increased confidence and motivation to continue with the sessions not only among the GFs but also among the GMs. One GM who had expressed her skepticism about the benefits of the GSP group sessions commented:

“After sharing my personal painful experiences, I really feel something in my life has changed. I used to lock myself in my room when I was annoyed and unhappy. Now I find myself sharing my meals with other people, something I used not to do” (Female GM who attended GSP sessions).

While the GMs participating in the GHE sessions similarly showed great interest in the topics discussed, their GFs did not observe marked changes in their emotions and behavior. Responses from GSP GFs and GMs indicate that the GSP intervention resulted in acquisition of a number of positive coping skills that potentially reduce depression. One GSP GM commented:

“I have learnt that keeping secrets that are hurting without sharing with others is very dangerous. I have also learnt that I am useful and important to self and others and I am hopeful about the future.”(Female GM who attended GSP sessions).

In addition, GSP participants reported less enacted stigma than GHE participants. Index scores for the loss of identity and status in the community factor generated from the factor analysis of the stigma scale were significantly lower among GSP than GHE participants (Mean difference between GHE and GSP = 0.44, p value = 0.05). Further, GMs responses provide evidence that the GSP intervention increased social connections and supports which are potent buffers against depression. One GM commented:

“Attending this group enabled me to unite with other people with the same problem whom I did not know before and we are now friends. (female GM)”

The GFs feedback on the last two sessions of the GSP intervention indicate that GMs were able to immediately put in practice the basic livelihood skills they had learnt. One GSP participant commented:

“Last year, at around this time, I had not paid fees for my children. Now, I no longer have a school fees problem. My relationship with my wife has improved” (Male GSP participant).

GFs reported that GMs were engaged in a variety of personal businesses and projects: buying and selling mangoes, oranges and sugar-canes, baking and selling goods in the markets, schools and on the streets. They reported on four livelihood groups that had been formed in Kitgum where the trial took place. *Kony Lawoti’* (Help Your Friend) group, *Yot Kom ayee Kwo’* (Health is Life) group, *Gen Ruoth’* (Trust in the Lord) group, and the *Lubanga Lakica’* (God is Merciful) group which had saved an equivalent of 58, 65, 146 and 77 USD respectively.

Depression treatment with GSP not only increased the ability to engage in income generating activities and acquire assets and savings but also appeared to promote healthy behaviors such as adherence to antiretroviral therapy. In the week prior to the 6 months follow-up interviews, a larger proportion of GSP GMs (96.0%) than GHE GMs (79.0%) were found not to have missed a dose of antiretroviral therapy on any day of the week (cluster adjusted χ^2^ = 3.65, p value = 0.05). Figure 1 summarizes the hypothesized causal mediating mechanisms between GSP and reduction in depression symptoms.

### Contextual Influences

Responses from both GFs and GMs revealed that factors which may have acted as facilitators or barriers of intervention delivery could be categorized as intrapersonal, cultural, gender based, institutional or program factors. Among intrapersonal factors, facilitators of intervention delivery included a keen interest and co-operation of the GMs in both GSP and GHE groups while barriers included personal vulnerabilities of GMs such as poor physical health which hindered attendance of group sessions while experience of intense emotional distress prolonged group meetings.

Two GFs reported:

“I was overwhelmed by the pathetic and deplorable situations experienced by the members who were sharing their personal stories. At this point, I felt that I needed a co-facilitator to help me handle the group session effectively and also to save time.” (Male GF for GSP sessions).

“One challenge which I experienced was about the breaking down of one group member who told her painful stories while crying and this delayed the meeting”. (Female GF for GSP sessions).

Suspected learning disabilities delayed progress of group sessions, and a few participants who were intoxicated with alcohol disrupted group proceedings. One GF reported:

“Some group members need a lot of explanation to understand the session. At the end of the session, I as a facilitator was very tired”. (Male GF for GSP sessions).

Table 4 summarizes other contextual influences as reported by both GFs and GMs in GSP or GHE sessions.

**Table 4:** Summary of the key themes from qualitative responses on contextual influences on intervention delivery

Group support psychotherapy (GSP)		Group HIV education (GHE)	
Group facilitators	Group members	Group facilitators	Group members
Facilitators of intervention delivery			
Intrapersonal factors		Intrapersonal factors	
Formal and informal training received	Keen interest and eagerness to learn	Some prior knowledge on HIV related issues	Keen interest and eagerness to learn Had some prior knowledge of the HIV topics
Prior mental health knowledge	Perceived GFs as supportive and respectful		Perceived GFs as supportive and respectful Adequate time was allocated to question and answer session
Program factors		Program factors	
Availability of intervention manual	Provision of transport allowance	Availability of intervention manual	Provision of transport allowance
Intervention content easy to follow	Provision of refreshments during sessions	Intervention content easy to follow	Provision of refreshments during sessions
Provision of financial incentive	GFs never missed a group session	Provision of financial incentive	GFs never missed a group session
Open communication channels with trainers	Intervention content culturally sensitive	Open communication channels with trainers	Intervention content culturally sensitive
Gender and cultural factors		Gender and cultural factors	
Allowed to elect their own group leader		Allowed to elect their own group leader	
Allowed to have an opening and closing ritual		Allowed to have an opening and closing ritual	
Allowed to give the group a traditional name		Allowed to give the group a traditional name	
Having gender specific sessions		Having gender specific sessions	
Barriers of intervention delivery			
Intrapersonal factors		Intrapersonal factors	
Impatience with slow learners and those intoxicated with alcohol	Poor time management	Feelings of guilt experienced when not able to teach coping skills yet GMs were emotionally unwell and some were intoxicated with alcohol	Some GMs were always intoxicated with alcohol
Limited knowledge on emotional self-care	Some GMs were always intoxicated with alcohol	Limited knowledge of ART medications	
Program factors		Program factors	
Intervention manual was not translated to the local language	Absence of writing material for GMs	Intervention manual was not translated to the local language	Some GMs were unhappy and disgruntled when they realized that they were not learning the same skills taught in GSP
	A few GMs complained of long distances between their homes and meeting venue	Sessions lacked information on Alcohol use problems	Absence of writing material for GMs who needed them
Financial incentive considered inadequate by the male GFs	Change of meeting venue without prior notice	Financial incentive considered inadequate by the male GFs	GMs not allowed to share their personal experiences
Lack of a co-facilitator increased work load	Time allocated for sharing personal problems not enough for GMs	Sessions not interactive and did not include practical sessions	No practical sessions given
Group size of 10–12 considered large given the multiple problems of PLWH	Meeting venue in mental health clinics were unpleasant to GMs	Lack of a co-facilitator increased work load	
Gender and cultural factors		Gender and cultural factors	
	GMs missed sessions due conflicting work schedules and burial attendances		Some husbands refused to grant permission to their wives to attend sessions Female GMs brought children to group sessions

## Discussion

The findings from this process evaluation complement our findings from the outcome evaluations of the randomized trial (Nakimuli-Mpungu et al. 2015). The reduction in depression and the increase in functioning levels observed in the trial can be explained by a number of processes observed in this study.

First, unlike previous studies of group psychotherapies for depression treatment in sub-Saharan Africa (Kaaya et al. 2013; Petersen et al. 2014), there was a high level of engagement with group support psychotherapy. Although the participant’s expectations of material gain were dispelled in the first session, the interventions reach, the dose delivered and received, and satisfaction indices were favorable suggesting that the intervention was highly feasible and acceptable to the target community. Such a high level of engagement, given the stigma attached to both HIV and mental illness, may surprise readers. Possible explanations for the success of GSP could be that the target community was involved in its development (Nakimuli-Mpungu et al. 2014a). Further, GSP was piloted prior to the trial and word had spread in the community about its benefits (Nakimuli-Mpungu et al. 2014b). In addition the intervention was culturally adapted.

Second, trained mental health workers were able to facilitate group support psychotherapy sessions following the intervention manual. Despite the personal, cultural and program barriers reported by the group facilitators, it is evident that their facilitation created a safe environment that allowed participants to acquire knowledge and skills which enabled them to experience a number of therapeutic processes associated with participation in group therapy.

For example, the group facilitators reported that all GSP participants had powerful emotional experiences referred to as catharsis in sessions 3 and 4. This experience is associated with the release of conscious or unconscious feelings followed by a feeling of great relief and it has been shown to result in immediate and long lasting changes (Brandler and Roman 2012).

Third, group facilitators reported that as sessions progressed, group members were able to provide feedback and give support to each other both during therapy and later in their livelihood groups. Participants reported on their perceived benefit of acquisition of skills to counsel others. This opportunity to help others through sharing experiences and giving advice, also referred to as altruism, can restore a sense of significance and increase self-esteem (Weinstein and Ryan 2010) that was one of the observed outcomes in the trial.

Fourth, the perceived benefit of increased social relationships indicates that development of socialization techniques—a powerful therapeutic factor—also took place during the group sessions. As hypothesized in our qualitative model of probable causal mechanisms, it is plausible that increased social connections and networks may have a direct effect on depression (Berkman et al. 2000). On the other hand, the stress-buffering model asserts that social support mitigates the relation between stressful life events and depression. Over the past four decades, a large body of research has consistently shown that the perception that one is accepted and valued in one’s interpersonal environment bolsters self-esteem, confidence, and self-efficacy, and promotes adaptive coping with stressors (Cobb 1976; Cohen and Wills 1985; Stein and Smith 2015). Therefore, it is also plausible that increased social support may have mitigated the impact of our targeted stressors such as maladaptive coping and stigma to reduce depression.

Lastly, our process data has shown that the sustained reduction in depression that we observed in the outcome evaluation of the trial (Nakimuli-Mpungu et al. 2015) was complimented by improved adherence to ART medications, improved functioning, reduction in alcohol intake, reduced enacted stigma and immediate initiation of income generating activities. GSP provided income generating skills as part of treatment for depression to alleviate poverty. Global mental health researchers have provided evidence that poor mental health interacts with poverty in a negative cycle and have called for the development of interventions that could break this cycle (Lund et al. 2011).

Data emerging from other randomized controlled trials of psychological interventions for depression among PLWH in developing countries are also promising (Chibanda et al. 2015). However, the absence of process evaluation data constraints the sustainable implementation of such interventions (Abasiubong et al. 2009; Chibanda et al. 2011; Kaaya et al. 2013; Petersen et al. 2014). Although randomized trials are regarded the gold standard for establishing the effectiveness of interventions, trial outcomes alone do not provide adequate information on how an intervention works and might be replicated in other similar settings (Craig et al. 2008). It is now recognized that process evaluation within trials are required to assess the quantity of the intervention delivered, whether the intervention was delivered as intended, whether the target study sample was the intended audience, how the intervention was delivered, clarify potential causal mechanisms and identify contextual influences on intervention delivery and outcomes (Oakley et al. 2006).

The results of our process evaluation suggest that GSP increased participation in income generating activities which, in turn, led to acquisition of assets and savings. The sustainable livelihoods framework that informs the development of GSP posits that the pursuit of livelihoods restores the dignity and independence of affected individuals (Carney 2003). It is plausible that the poverty- or stigma-reduction effects of the livelihood intervention previously documented by other researchers (Tsai et al. 2013, 2016; Lund 2015) may explain the sustained reduction in depression and increase in functionality which was demonstrated in the trial outcomes published earlier (Nakimuli-Mpungu et al. 2015).

The findings from both the RCT process and outcome evaluations of GSP support this intervention as a first-line treatment for depression in resource poor settings, and an intervention that may be able to break the negative cycle linking depression and poverty. However, these results should be viewed within the context of the following limitations. First, it is recommended that implementers of an intervention be different than the evaluators of the intervention (Craig et al. 2008). However, due to limited capacity in our low-resource setting it was not possible to have a separate implementation and evaluation team. In order to limit the risk that process data interpretation would be biased by knowledge of the trial outcomes, process data was collected before trial data were analyzed.

Second, customary methods of supervision were not possible due to limited capacity and resources. To mitigate this limitation, we provided both theoretical and practical training sessions, financial incentives, and videotaped training sessions which they could refer to after the workshop. We also piloted the delivery of group sessions before facilitating group sessions in the trial. The GFs were asked to record and report on what transpired in group sessions and advice was relayed back via email and phone calls.

Third, our study sample was primarily of Luo ethnicity and the results may not directly generalize to the different ethnic populations in the northern region of Uganda. Greater representation from other ethnic groups could help insure generalizability of study findings to the region. Lastly, the adherence measurement method used in this study may overestimate the adherence rate to antiretroviral medication. However, other adherence measures described in the literature may also overestimate the adherence rate. For example, pharmacy-based measures rely on the assumption that the patients took all their medications during the interval between clinic visits, which may not be the case. Further, to date there is no established gold standard to measure ART adherence (Bangsberg et al. 2001).

Despite these limitations, there are important lessons learned from the process data we obtained. Participants from GSP groups reported that the intervention had helped them reduce their alcohol consumption. This finding is not surprising because previous researchers have shown that depression treatment with cognitive behavioral based interventions in persons with depression and alcohol use problems usually reduces the frequency of drinking (Moak et al. 2003).

Having to deal with participants who were intoxicated with alcohol during the group sessions was a challenge to both facilitators and trial participants. The trial data showed that hazardous alcohol consumption was significantly associated with attrition from the study. Therefore, it may be necessary to determine the stage of readiness for behavior change where an individual with a drinking problem is before allowing them to participate in GSP sessions. Previous research has shown that individuals at the pre-contemplation stage of the Proshaska model of behavior change may not be responsive to interventions (Prochaska and DiClemente 1986).

Also, we learned that the process of facilitation of the GSP group sessions was stressful to the facilitators, indicating that co-facilitation of groups may be necessary, especially for larger groups. Further, there is need for the facilitators to have competency in self-awareness and self care, a subject that we did not address during training, so that they can cope better with the challenges of delivering GSP.

One implication of the findings of this study is that it is possible to increase capacity for first-line depression treatments such as GSP in low resource rural areas across the globe using health professionals other than highly trained mental health providers. The fact that GSP participants reported counseling others in the villages that had similar problems, suggests that these trained health professionals can then train lay people to provide depression care in their communities. This shifting of mental health related tasks from health professionals to para-professionals or non-health professionals (lay health workers) has been documented in non-HIV populations as well (Araya et al. 2006; Rahman et al. 2008; Patel et al. 2010; Bolton et al. 2007). However, since mental health has not been integrated into HIV care in sub-Saharan Africa, little is known about the effectiveness of task shifting of mental health related services for this population.

We now have evidence that specialists at tertiary institutions can train mid-level mental health workers to effectively deliver GSP (Nakimuli-Mpungu et al. 2015). Going forward, we plan to expand capacity at primary healthcare (PHC) centers in three districts in northern Uganda to diagnose and treat depression in PLWH receiving HIV services. Strategies to achieve this plan include developing tailored training curricula that can effectively equip non-specialized health workers at PHC centers with competencies required to not only recognize depression and implement GSP, but also to train lay health workers affiliated with their PHC centers to acquire competencies required to deliver GSP to PLWH who need it in the community, thereby making first line treatments more widely accessible and sustainable.
